# Case Report: Postoperative pulmonary embolism causing obstructive shock and circulatory collapse after gynecologic surgery: successful rescue with VA-ECMO

**DOI:** 10.3389/fcvm.2026.1912489

**Published:** 2026-09-16

**Authors:** Nguyen Kim Long, Huynh Quang Dai, Cao Hoai Tuan Anh, Pham Duc Dat, Khau Minh Tuan, Tran Huy Nhat, Nguyen Huu Tin, Vu Phan Anh Tuan, Nguyen Minh Nhut, Nguyen Truong Hai, Dinh Than Thuong

**Affiliations:** 1 115 People's Hospital, Ho Chi Minh City, Vietnam; 2 School of Medicine, University of Medicine and Pharmacy at Ho Chi Minh City, Ho Chi Minh City, Vietnam

**Keywords:** extracorporeal membrane oxygenation, gynecologic surgery, obstructive shock, pulmonary embolism (PE), venous thromboembolism

## Abstract

Very-high-risk pulmonary embolism (PE) occurring within hours after major surgery presents a therapeutic dilemma because urgent reperfusion may be required at precisely the time when bleeding risk is greatest. We describe a 45-year-old woman who developed catastrophic bilateral PE approximately two hours after elective gynecologic surgery consisting of myomectomy and ovarian endometriotic cyst excision. Shortly after surgery, she experienced abrupt respiratory failure, profound hypotension, and rapidly progressive circulatory collapse. On arrival at a tertiary intensive care unit, arterial blood gas analysis revealed severe respiratory acidosis (pH 7.019; PaCO_2_ 82 mmHg), and the patient remained unstable despite mechanical ventilation, high-dose vasopressors, and conventional resuscitative measures. Because of refractory obstructive shock and the prohibitive hemorrhagic risk associated with recent pelvic surgery, emergent venoarterial extracorporeal membrane oxygenation (VA-ECMO) was instituted before definitive radiological confirmation. Computed tomography pulmonary angiography subsequently demonstrated extensive bilateral pulmonary arterial thrombosis involving both proximal and distal branches. Management was further complicated by ongoing postoperative bleeding during mandatory anticoagulation for ECMO support, requiring repeated transfusion of blood products and frequent adjustment of anticoagulant therapy. During recovery, the patient also developed residual left popliteal deep vein thrombosis, postoperative inguinal hematoma, and surgical-site infection caused by extended-spectrum β-lactamase-producing Escherichia coli. Despite these challenges, she was successfully weaned from ECMO and mechanical ventilation, transitioned to oral rivaroxaban, and discharged without neurological sequelae. This case illustrates the complexity of managing acute PE when life-saving reperfusion strategies must be balanced against a substantial risk of postoperative bleeding. It also adds to the limited literature describing successful use of VA-ECMO as a bridge to recovery in immediate postoperative PE complicated by obstructive shock and circulatory collapse.

## Introduction

Acute pulmonary embolism (PE) remains one of the leading causes of cardiovascular death and is the most serious presentation within the spectrum of venous thromboembolic disease (1–3) [1–3]. While the majority of patients are hemodynamically stable at presentation, a small proportion develop high-risk PE accompanied by persistent hypotension, obstructive shock, or cardiac arrest (1, 4) [1, 4]. Mortality rises sharply once circulatory failure occurs and remains particularly high in patients requiring resuscitation (1, 4, 5) [1, 4, 5]. The immediate threat is acute right ventricular failure resulting from a sudden increase in pulmonary vascular resistance, with subsequent reduction in left ventricular filling and systemic perfusion (5, 6) [5, 6]. Rapid restoration of pulmonary blood flow is therefore central to management. Current ESC guidelines recommend urgent reperfusion using systemic thrombolysis, catheter-based intervention, or surgical embolectomy in patients with high-risk PE (1). However, therapeutic decision-making becomes considerably more complex when the embolic event occurs shortly after major surgery, where the need for reperfusion must be weighed against the risk of severe postoperative bleeding.

Venous thromboembolism is a recognized complication of major gynecologic and pelvic surgery because all components of Virchow's triad may be present during the perioperative period (2, 7) [2, 7]. In patients who progress to refractory obstructive shock or circulatory arrest, VA-ECMO can provide temporary cardiopulmonary support while anticoagulation or reperfusion strategies are considered (8–10) [8, 9]. Nevertheless, the evidence base remains limited, and questions remain regarding the optimal timing of ECMO initiation, patient selection, and anticoagulation management in the setting of active postoperative bleeding (8–10) [8, 9]. Reports describing very-high-risk PE occurring within only a few hours after gynecologic surgery are uncommon, and successful treatment with VA-ECMO in this context has been infrequently documented. We report a patient who developed catastrophic bilateral PE shortly after myomectomy and ovarian endometriotic cyst excision, leading to obstructive shock and circulatory arrest, and who survived following emergent VA-ECMO despite significant postoperative hemorrhagic complications.

## Case presentation

A 45-year-old woman was admitted to Hung Vuong Hospital for elective gynecologic surgery, including excision of an ovarian endometriotic cyst and myomectomy for symptomatic uterine fibroids. Available medical records revealed no documented history of venous thromboembolism, chronic cardiopulmonary disease, malignancy, or known thrombophilia. Information regarding body mass index, formal preoperative venous thromboembolism risk assessment (including Wells and Caprini scores), perioperative pharmacological or mechanical thromboprophylaxis, hormonal therapy exposure, and family history of thrombotic disorders was not available from the retrieved records. Because the patient was transferred emergently from another institution after surgery, the original operative records were unavailable. Consequently, the surgical approach (open vs. laparoscopic) and the exact intraoperative positioning could not be reliably determined or reconstructed retrospectively.

The surgical procedure was completed without any reported intraoperative hemodynamic instability. No documented intraoperative cardiorespiratory complications were identified in the available operative records. However, approximately two hours after surgery, the patient developed sudden-onset respiratory distress accompanied by profound hypotension and rapidly progressive circulatory collapse. The transferred records did not provide further details regarding the patient's presenting symptoms immediately before clinical deterioration, such as chest pain, the severity of dyspnea, or syncope, because the patient's condition deteriorated rapidly and emergency transfer was prioritized. Her clinical condition deteriorated within minutes, with features suggestive of obstructive shock and impending cardiac arrest. Given the high clinical suspicion of acute PE, she was emergently transferred to the Intensive Care Unit of 115 People's Hospital, a tertiary referral center with extracorporeal life support capability. Because the patient was transferred emergently from another institution during ongoing resuscitation, the transferred records did not clearly document whether complete cardiopulmonary arrest occurred before transfer, whether external cardiopulmonary resuscitation was performed, or the duration of any low-flow period.

Upon arrival, the patient was critically ill. She exhibited severe acute respiratory failure and refractory shock requiring immediate advanced resuscitative measures. Arterial blood gas analysis demonstrated profound respiratory acidosis, with a pH of 7.019 and a PaCO₂ of 82 mmHg, indicating severe ventilatory failure and inadequate gas exchange. Endotracheal intubation and invasive mechanical ventilation were promptly instituted. Despite aggressive fluid resuscitation and escalating doses of norepinephrine and epinephrine, hemodynamic instability persisted, raising concern for rapidly progressive right ventricular failure secondary to massive pulmonary vascular obstruction. Initial laboratory investigations demonstrated markedly elevated D-dimer levels. Coagulation testing was initially within the therapeutic target range before subsequent anticoagulation-related abnormalities developed. No evidence of pre-existing renal dysfunction was identified at admission.

Because of ongoing circulatory collapse despite conventional resuscitation, venoarterial extracorporeal membrane oxygenation (VA-ECMO) was established emergently as a rescue circulatory support strategy. Systemic anticoagulation with unfractionated heparin was initiated according to institutional ECMO protocols. Following ECMO implantation, systemic perfusion improved sufficiently to permit completion of definitive diagnostic investigations. Continuous anticoagulation with unfractionated heparin was administered throughout extracorporeal support, with serial adjustment according to coagulation parameters and bleeding severity. During the initial ECMO course, the patient remained deeply sedated with propofol, midazolam, and fentanyl and required invasive mechanical ventilation together with high-dose vasopressor support. Progressive hemodynamic stabilization was observed over the subsequent days, allowing gradual reduction of vasopressor requirements and stepwise ECMO weaning. Extracorporeal support was successfully discontinued between hospital days 5 and 7 after recovery of native cardiopulmonary function, and no major ECMO circuit-related complications were documented.

Given the complexity of the clinical situation, management decisions were made through multidisciplinary discussion involving intensivists, cardiovascular specialists, anesthesiologists, gynecologic surgeons, hematology/transfusion support, and imaging/interventional teams as appropriate. Because the patient presented with refractory obstructive shock only hours after major pelvic surgery, the team jointly weighed the competing risks of immediate reperfusion, ongoing postoperative bleeding, anticoagulation required for VA-ECMO, and the feasibility of catheter-based or surgical interventions. Consensus was reached to proceed with emergent VA-ECMO and carefully titrated unfractionated heparin while closely monitoring both thrombotic and hemorrhagic complications.

Bedside transthoracic echocardiography demonstrated marked right ventricular dilation with regional right ventricular free-wall hypokinesia and preserved apical contractility (McConnell's sign), findings highly suggestive of acute right ventricular pressure overload secondary to massive PE (Figure 1).

**Figure 1:**
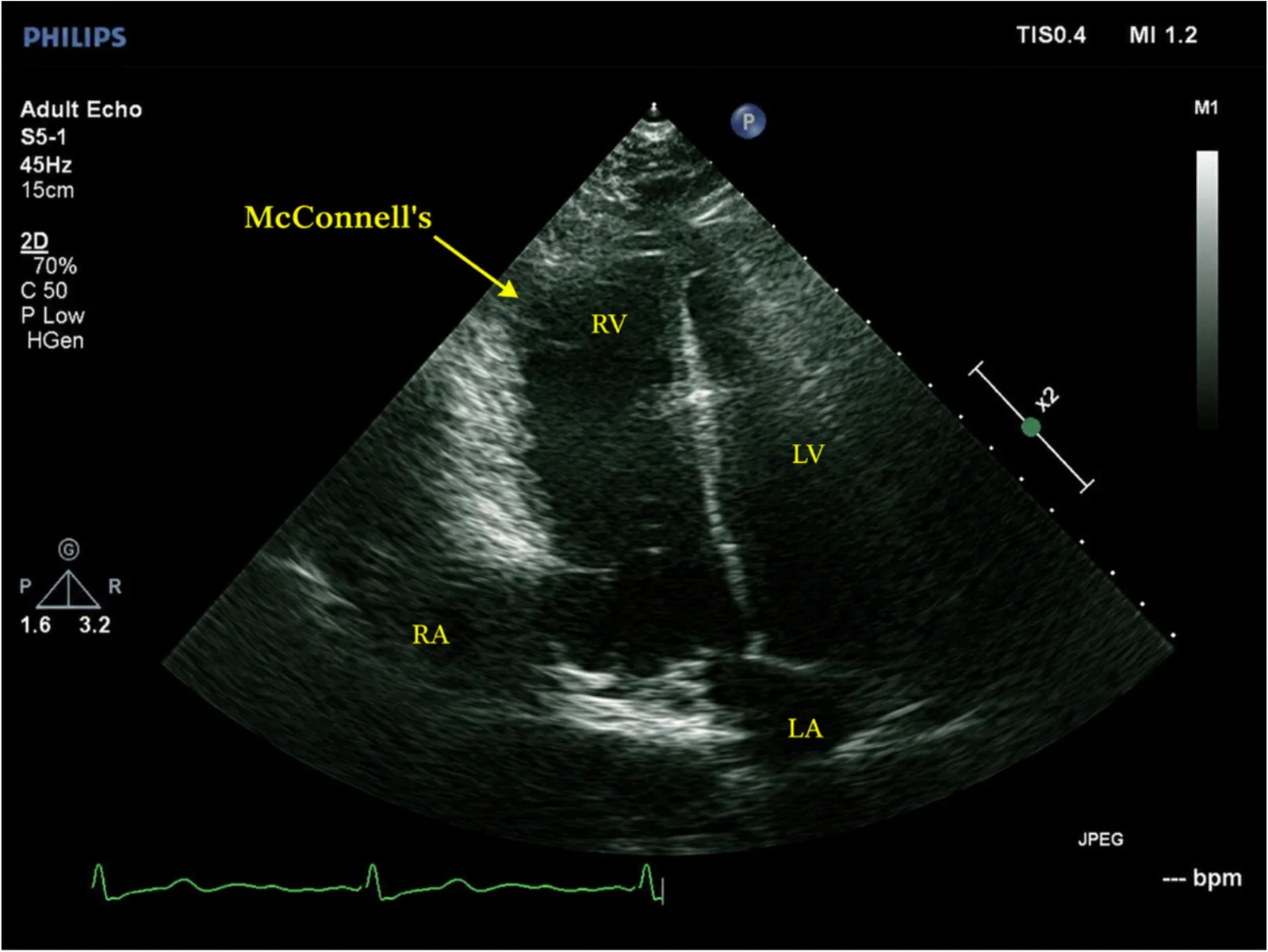
Bedside transthoracic echocardiography demonstrating mcConnell's sign in acute high-risk PE. Apical four-chamber view showing right ventricular enlargement with akinesia of the mid-free wall and preserved apical contractility (McConnell's sign), consistent with acute right ventricular pressure overload secondary to massive PE. RV: right ventricle; LV: left ventricle; RA: right atrium; LA: left atrium.

Computed tomography pulmonary angiography confirmed extensive acute bilateral PE involving both proximal pulmonary arteries and multiple distal branches (Figure 2). Additional computed tomography of the abdomen demonstrated postoperative intra-abdominal fluid accumulation and findings suspicious for a pelvic hematoma. Findings were also suggestive of systemic venous congestion, including poor enhancement of the inferior vena cava and hepatic veins, heterogeneous hepatic enhancement, and small-volume ascites (Figure 3).

**Figure 2:**
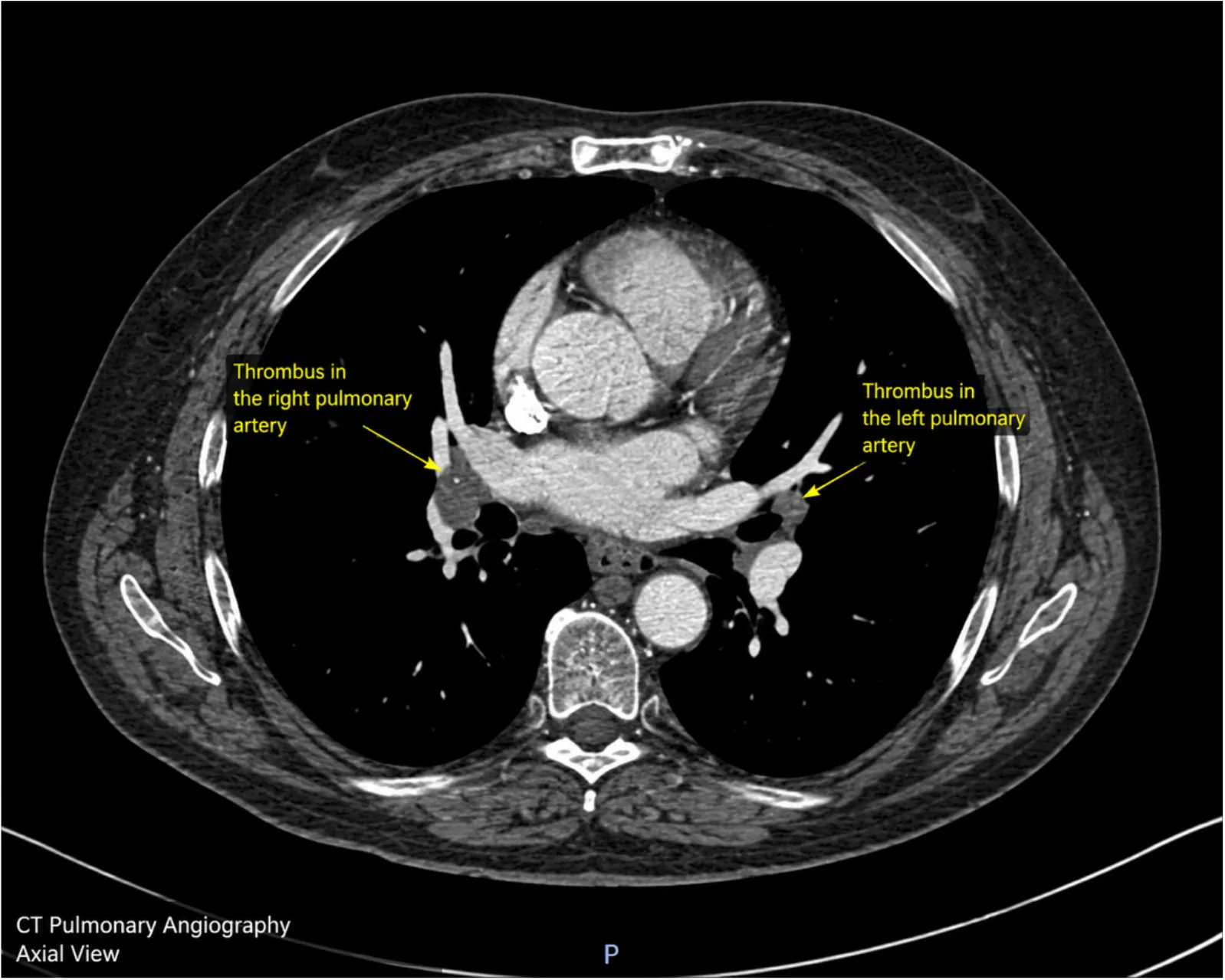
Computed tomography pulmonary angiography demonstrating acute bilateral PE involving the main pulmonary arterial branches. Axial CTPA image showing intraluminal filling defects within both the right and left pulmonary arteries, consistent with acute bilateral PE.

**Figure 3:**
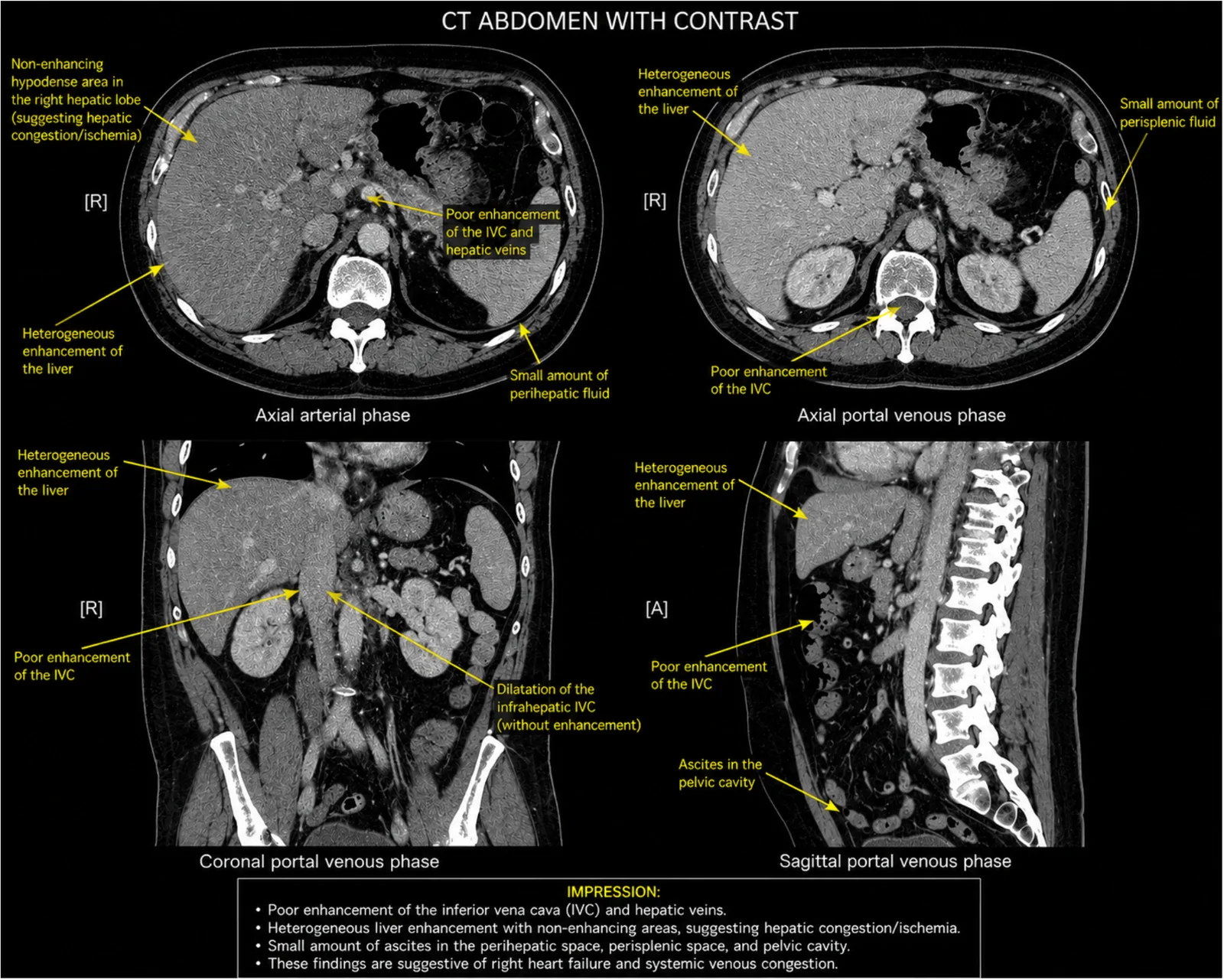
Contrast-enhanced abdominal computed tomography demonstrating systemic venous congestion associated with acute right ventricular failure. Multiplanar abdominal CT images showing poor enhancement of the inferior vena cava and hepatic veins, heterogeneous hepatic parenchymal enhancement suggestive of hepatic congestion/ischemia, and small-volume ascites. These findings are consistent with systemic venous congestion secondary to acute right-sided circulatory failure caused by massive PE.

The overall clinical picture was consistent with very-high-risk PE complicated by obstructive shock and peri-arrest circulatory failure. The thrombotic burden involved both proximal and distal pulmonary arterial branches bilaterally, consistent with a large-volume embolic event (Figure 4). Lower-lobe atelectatic changes were also noted on thoracic imaging.

**Figure 4:**
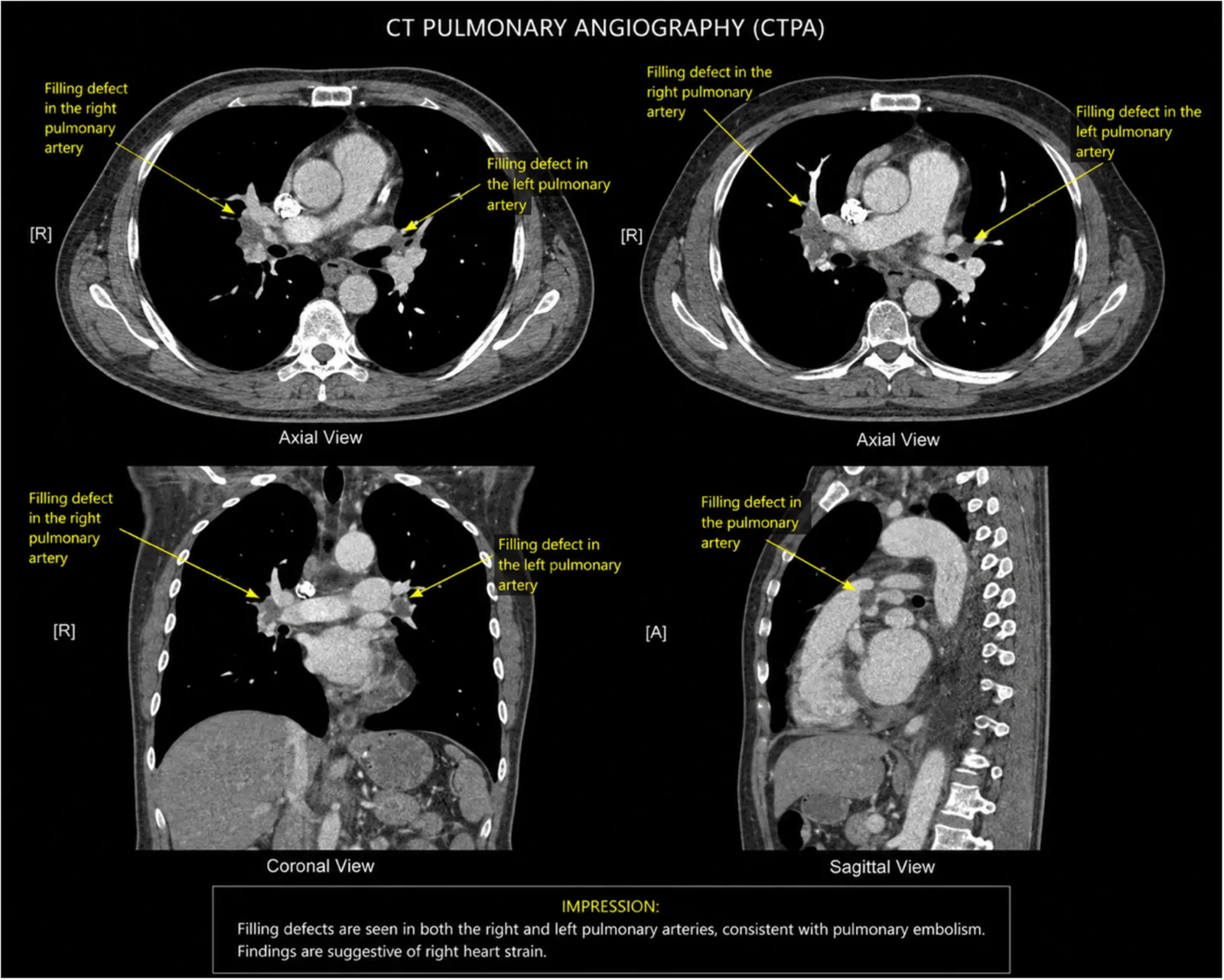
Multiplanar computed tomography pulmonary angiography demonstrating extensive bilateral pulmonary arterial thromboembolic burden. Axial, coronal, and sagittal CTPA reconstructions demonstrating multiple intraluminal filling defects involving the right and left pulmonary arteries and their segmental branches, confirming extensive acute bilateral PE.

During the first several days of intensive care support, management was complicated by the competing risks of thrombosis and hemorrhage. Continuous anticoagulation was required to maintain ECMO circuit patency and prevent thrombus propagation; however, clinically significant postoperative bleeding developed simultaneously. Persistent oozing was observed from the abdominal surgical site, drainage catheters, and ECMO cannulation sites. Coagulation testing demonstrated marked anticoagulation, with activated partial thromboplastin time (aPTT) values exceeding 140 s. Concurrently, hemoglobin concentrations declined to approximately 8.0 g/dL, necessitating repeated transfusions of packed red blood cells, fresh frozen plasma, and cryoprecipitate. Anticoagulation intensity was therefore continuously adjusted according to serial coagulation parameters and bleeding severity.

The patient remained sedated with propofol, midazolam, and fentanyl during the acute phase while receiving lung-protective mechanical ventilation and full VA-ECMO support. Over the ensuing days, gradual improvement in tissue perfusion and respiratory status was observed. Vasopressor requirements progressively decreased, arterial blood gas parameters improved, and serial lactate measurements demonstrated a favorable trend. Serial arterial blood gas analyses showed progressive correction of acid–base disturbances, while renal function remained stable throughout the period of extracorporeal support. Only mild transient elevations of hepatic transaminases were observed.

As cardiopulmonary function recovered, ECMO flow was progressively reduced and weaning trials were successfully performed. Between hospital days 5 and 7, the patient regained consciousness following withdrawal of sedation, demonstrated preserved neurological responsiveness, and tolerated discontinuation of extracorporeal support. VA-ECMO was subsequently removed without major procedural complications. Mechanical ventilation was successfully discontinued thereafter, and the patient was extubated to supplemental oxygen delivered via nasal cannula. Following ECMO decannulation and extubation, oxygenation remained satisfactory, with peripheral oxygen saturation maintained between 96% and 98% on low-flow oxygen therapy.

Following recovery from the initial life-threatening phase, additional complications emerged. The patient developed intermittent fever and purulent discharge from the surgical wound. Laboratory evaluation demonstrated elevated inflammatory biomarkers, including a C-reactive protein concentration of 31.22 mg/L and a procalcitonin level of 0.780 ng/mL. Although repeated blood cultures remained negative, microbiological analysis of wound and intra-abdominal drainage specimens yielded extended-spectrum *β*-lactamase (ESBL)-producing *Escherichia coli*. Antimicrobial therapy was tailored according to susceptibility testing, with meropenem administered as definitive treatment. Daily wound irrigation, local debridement, and meticulous wound care were performed to achieve source control.

A duplex ultrasonographic examination performed on June 16, 2025, identified residual deep venous thrombosis involving the left popliteal vein, supporting a venous thromboembolic origin of the pulmonary embolic event (Figure 5). Additional soft-tissue ultrasonography revealed a left inguinal fluid collection measuring 97 × 35 mm, compatible with postoperative hematoma or seroma adjacent to the previous cannulation region. As bleeding gradually resolved, anticoagulation therapy was sequentially transitioned from intravenous unfractionated heparin to low-molecular-weight heparin and subsequently to oral rivaroxaban for long-term secondary prevention of venous thromboembolism.

**Figure 5:**
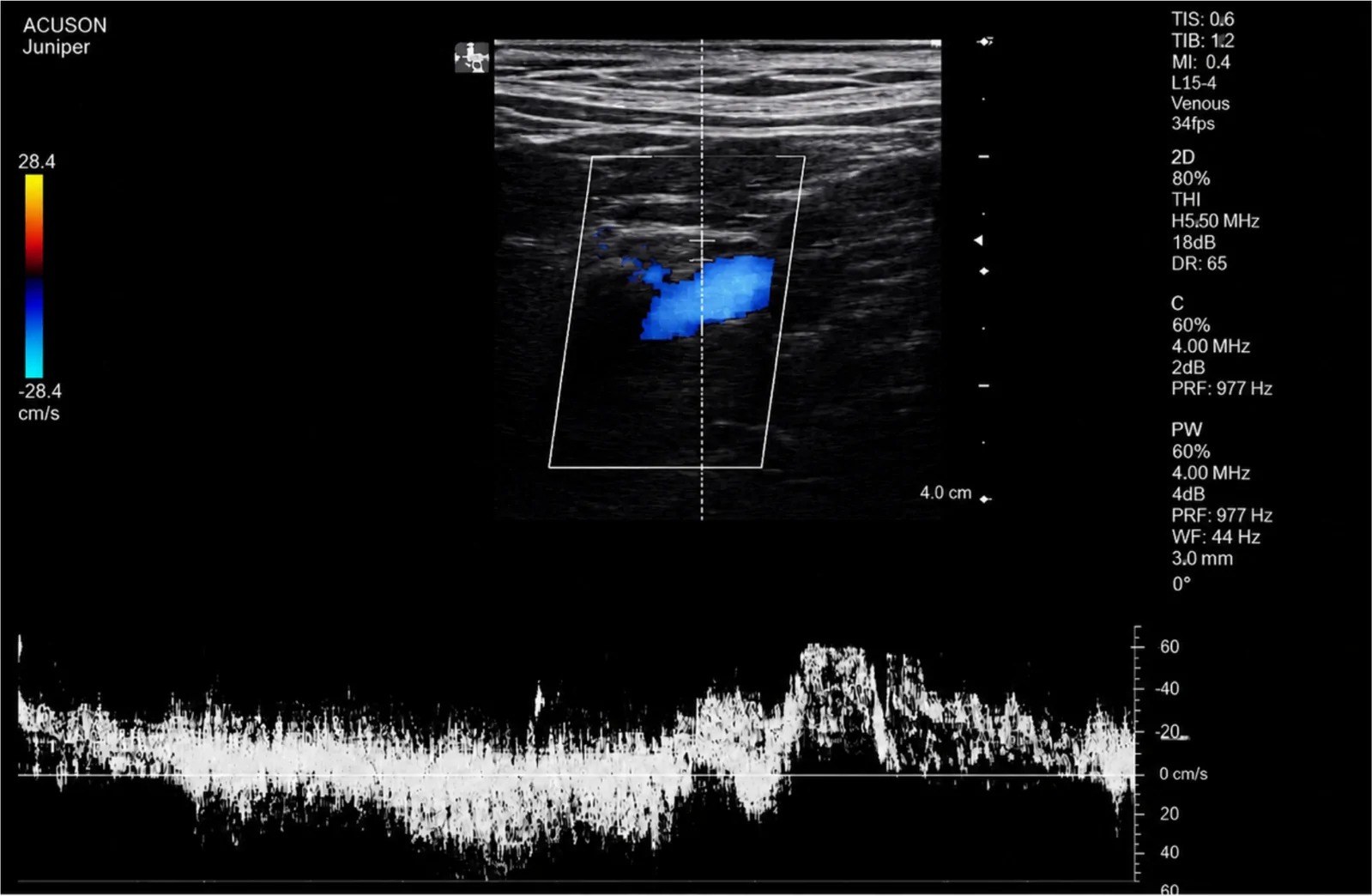
Duplex venous ultrasonography demonstrating extensive left lower-extremity deep vein thrombosis. Duplex ultrasound showing thrombotic occlusion involving the left popliteal vein, supporting the venous thromboembolic source of PE.

The patient continued to improve clinically throughout the remainder of her hospitalization. Supplemental oxygen was discontinued, inflammatory markers progressively decreased, and hematologic as well as biochemical parameters stabilized. At discharge on June 24, 2025, she was fully conscious, breathing comfortably on room air, hemodynamically stable, ambulatory, and able to tolerate oral intake. Surgical wounds had healed satisfactorily, and no evidence of recurrent thromboembolic events was observed. She was discharged on rivaroxaban therapy with scheduled outpatient follow-up for ongoing assessment of venous thromboembolism recurrence, anticoagulation management, and potential long-term sequelae of massive PE. At discharge, blood pressure remained stable at approximately 110–120/60–70 mmHg, heart rate ranged from 80 to 90 beats/min, and the patient was able to ambulate independently without supplemental oxygen.

## Discussion

This report describes a case of very-high-risk postoperative pulmonary embolism that developed approximately two hours after gynecologic surgery and progressed rapidly to obstructive shock and circulatory collapse. Although pelvic surgery is a recognized risk factor for venous thromboembolism, clinically apparent PE is more often diagnosed several days after the procedure rather than during the immediate postoperative period (10, 11). The extremely short interval between surgery and clinical deterioration raises the possibility that thrombus formation had already begun before or during the operation and embolized shortly thereafter. Such a sequence is biologically plausible in the setting of pelvic surgery, where endothelial injury, venous stasis, and transient postoperative hypercoagulability may coexist and reinforce one another (10, 12). In addition, prolonged operative positioning, particularly lithotomy or Trendelenburg positioning when used during gynecologic surgery, may further reduce lower-extremity venous return and contribute to venous stasis, potentially increasing the likelihood of embolization from an existing lower-extremity thrombus. Although detailed operative records describing the surgical approach and intraoperative positioning were unavailable in this transferred case, these mechanisms remain biologically plausible contributors to the very short interval between surgery and symptom onset observed in this patient. For the treating clinician, the key implication is that PE cannot be excluded solely on the basis of timing; sudden respiratory compromise and unexplained shock in the first postoperative hours should prompt immediate consideration of this diagnosis.

The initial physiological derangement was striking. Acute PE produces hemodynamic instability through abrupt elevation of pulmonary vascular resistance, leading to acute right ventricular pressure overload, reduced left ventricular filling, and ultimately systemic circulatory failure (13, 14). Previous studies have shown that obstruction of more than 30%–50% of the pulmonary vascular bed may be sufficient to induce significant right ventricular dysfunction, whereas larger embolic burdens may precipitate obstructive shock or cardiac arrest (13). In this patient, the arterial blood gas profile was notable for severe respiratory acidosis (pH 7.019; PaCO₂ 82 mmHg). This finding contrasts with the hypocapnia more commonly encountered in acute PE, where compensatory hyperventilation is typically present (14). Marked hypercapnia is uncommon during the early phase of acute PE and generally reflects advanced physiological decompensation rather than the embolic event itself. In our patient, severe hypercapnia most likely resulted from peri-arrest physiology associated with profound obstructive shock, markedly reduced pulmonary perfusion, ineffective alveolar ventilation, and impaired carbon dioxide elimination during rapidly progressive circulatory collapse. The requirement for immediate endotracheal intubation, invasive mechanical ventilation, and high-dose vasopressor support on arrival further suggests that arterial blood gas analysis was obtained at a late stage of physiological deterioration rather than during the initial presentation of PE. Together with the profound hemodynamic instability, this degree of hypercapnia suggested a critically reduced physiological reserve and a high probability of imminent cardiovascular collapse. Similar observations have been associated with advanced right ventricular failure and adverse outcomes in patients with massive PE (1).

Several alternative causes of sudden postoperative respiratory and hemodynamic collapse were considered during the initial evaluation. Potential differential diagnoses included anesthetic-related hypoventilation or residual neuromuscular blockade, aspiration, acute upper airway obstruction, acute myocardial infarction, hemorrhagic shock, anaphylaxis, and septic or other distributive shock. However, these possibilities became less likely after bedside transthoracic echocardiography demonstrated acute right ventricular pressure overload with McConnell's sign, while subsequent computed tomography pulmonary angiography confirmed extensive bilateral pulmonary embolism. The later identification of residual left popliteal deep vein thrombosis further supported venous thromboembolism as the underlying etiology.

The pivotal management decision was the early deployment of VA-ECMO following multidisciplinary discussion. In patients with catastrophic postoperative pulmonary embolism, therapeutic decisions frequently require close collaboration among intensivists, cardiologists, anesthesiologists, surgeons, hematologists, and imaging/interventional specialists because life-saving reperfusion must be balanced against the competing risks of postoperative hemorrhage and anticoagulation. In the present case, this multidisciplinary approach facilitated a unified treatment strategy that prioritized immediate circulatory stabilization with VA-ECMO while allowing continuous reassessment of bleeding control, anticoagulation intensity, potential advanced reperfusion therapies, infection management, and long-term prevention of recurrent venous thromboembolism. Current guidelines recommend systemic thrombolysis as first-line reperfusion therapy for high-risk PE accompanied by shock or persistent hypotension (15). Alternative approaches include catheter-directed thrombectomy, surgical embolectomy, and selected use of inferior vena cava filters in specific clinical circumstances. The potential role of an inferior vena cava (IVC) filter also warrants consideration in this clinical context. Although residual left popliteal deep vein thrombosis was identified during recovery, therapeutic anticoagulation had already been initiated for ECMO support and was maintained throughout the acute phase with careful adjustment according to bleeding severity. Therefore, anticoagulation was not absolutely contraindicated or permanently discontinued. Under these circumstances, routine IVC filter placement was not considered indicated because therapeutic anticoagulation could be maintained. Contemporary guidelines recommend restricting IVC filter placement to carefully selected patients with acute venous thromboembolism who have an absolute contraindication to anticoagulation or recurrent embolism despite adequate anticoagulant therapy. Routine IVC filter placement is not recommended when therapeutic anticoagulation can be safely maintained. However, the therapeutic options available in guidelines are not always applicable to the patient in front of the clinician. In the present case, major pelvic surgery had been completed only hours earlier, and significant postoperative bleeding subsequently developed even during conventional anticoagulant therapy. Under these circumstances, immediate thrombolysis would have entailed a substantial risk of uncontrolled hemorrhage (16). VA-ECMO was therefore chosen to stabilize circulation and maintain end-organ perfusion while avoiding early exposure to fibrinolytic treatment. This strategy is consistent with the growing use of extracorporeal support in carefully selected patients with refractory obstructive shock due to acute PE, particularly when conventional reperfusion options are limited by bleeding concerns or cannot be implemented immediately (17, 18).

The role of ECMO in high-risk PE is still unsettled. Unlike systemic thrombolysis, catheter-directed therapy, or surgical embolectomy, ECMO does not remove the obstructing clot (8, 18). Its value is instead physiological: it buys time by restoring systemic perfusion, reducing dependence on escalating catecholamines, unloading the failing cardiopulmonary circulation, and preserving end-organ oxygen delivery while anticoagulation, endogenous fibrinolysis, or delayed reperfusion strategies take effect (17, 18). In the multicenter European series by Meneveau and colleagues, survival to hospital discharge was approximately 60% among patients treated with ECMO for high-risk PE, despite the critical severity of the cohort (9). Other syntheses have also suggested a survival signal when ECMO is instituted before irreversible multiorgan failure, particularly in patients presenting with cardiac arrest or refractory shock (16, 18). Yet the available data remain observational, heterogeneous, and highly dependent on timing, patient selection, and whether ECMO is used alone or in combination with catheter-based or surgical reperfusion. In the present case, ECMO should therefore be viewed not as definitive clot therapy, but as a rescue platform that maintained perfusion when thrombolysis was judged unsafe and immediate definitive reperfusion was not pursued.

The most difficult bedside problem after cannulation was not whether anticoagulation was indicated, but how much anticoagulation the patient could tolerate. ECMO required continuous anticoagulation to preserve circuit patency and limit further thrombus propagation, whereas the fresh pelvic surgical field, abdominal drains, and cannulation sites created an immediate bleeding hazard (17, 19). This conflict became clinically apparent when the activated partial thromboplastin time exceeded 140 s, accompanied by persistent wound and drain-site bleeding and a hemoglobin decline to approximately 8.0 g/dL, requiring packed red blood cells, fresh frozen plasma, and cryoprecipitate. Complete interruption of anticoagulation was not a realistic option because the thrombotic burden remained substantial. Management therefore depended on repeated titration rather than a fixed protocol: serial coagulation assessment, visual assessment of bleeding, transfusion support, and close communication between intensivists, surgeons, hematologists, and perfusionists. This narrow margin between circuit thrombosis and postoperative hemorrhage is the central practical lesson of the case. More broadly, this experience highlights the importance of multidisciplinary decision-making throughout the patient's clinical course, as successful management depended not on a single intervention but on continuous coordination among critical care, cardiovascular, surgical, hematology, anesthesiology, and imaging teams as clinical priorities evolved. Recent evidence from the RIETE Registry further highlights that bleeding during anticoagulation for venous thromboembolism is a dynamic and multifactorial process rather than a uniform treatment-related complication (20). In a large contemporary cohort of more than 19,000 patients with acute venous thromboembolism, bleeding events occurred early during the first 90 days of anticoagulant therapy, supporting the concept that bleeding risk should be reassessed continuously throughout treatment rather than assumed to remain constant over time (20). Although that study specifically investigated low LDL-cholesterol as a potential marker of bleeding susceptibility, it also reinforces the broader principle that bleeding risk among anticoagulated patients is heterogeneous and requires individualized clinical assessment (20). In our patient, multiple bleeding risk factors converged simultaneously, including very recent major pelvic surgery, fresh surgical wounds and drains, mandatory anticoagulation for VA-ECMO, multiple vascular cannulation sites, and the need for repeated transfusion support. These observations emphasize the importance of dynamic, individualized reassessment rather than reliance on fixed anticoagulation protocols when balancing thrombotic and hemorrhagic risks in critically ill patients.

Our patient subsequently developed two clinically important complications: residual left popliteal deep venous thrombosis and postoperative wound infection caused by ESBL-producing Escherichia coli. These events occurred after successful reversal of obstructive shock and illustrate that the clinical trajectory of massive PE extends well beyond initial hemodynamic stabilization. Detection of residual deep venous thrombosis during recovery provided objective evidence supporting the venous source of embolization and reinforced the need for continued therapeutic anticoagulation despite the recent history of significant bleeding (20). At the same time, prolonged ICU stay, invasive mechanical ventilation, extracorporeal support, repeated transfusions, and multiple vascular access devices likely increased susceptibility to secondary infection (19). The development of an ESBL-producing organism further complicated management because treatment priorities shifted from circulatory rescue toward infection control, wound management, and prevention of recurrent thromboembolic events. This sequence of complications reflects a reality frequently encountered in critically ill survivors of massive PE, in whom successful resuscitation represents only the beginning of a prolonged and complex recovery process.

Published reports describing ECMO-supported massive PE after gynecological surgery remain limited (8, 9, 16, 18). Rogers and colleagues reported successful use of ECMO in a postoperative gynecological patient with massive PE; however, the present case differed in several clinically relevant aspects, including the exceptionally short interval between surgery and circulatory collapse, the coexistence of major postoperative bleeding requiring ongoing anticoagulation, and the occurrence of both residual DVT and multidrug-resistant surgical-site infection during recovery (11). Another notable feature is that favorable clinical recovery was achieved without systemic thrombolysis, catheter-directed reperfusion, or surgical embolectomy, despite the severity of the initial presentation. Nevertheless, interpretation of this experience requires caution. As a single-patient report, it cannot determine the relative contribution of ECMO to outcome or support broad therapeutic recommendations. Several potentially informative variables, including detailed operative records (such as the surgical approach and intraoperative positioning), detailed echocardiographic measurements, cardiac biomarker profiles, whether cardiopulmonary resuscitation was performed, the exact duration of any low-flow period, and detailed ECMO flow parameters, were unavailable from the transferred medical records. Formal preoperative venous thromboembolism risk assessment (including Wells and Caprini scores) and documentation of perioperative pharmacological or mechanical thromboprophylaxis were also unavailable. In addition, long-term follow-up data regarding recurrent venous thromboembolism, chronic thromboembolic pulmonary hypertension, functional recovery, and quality of life were not available at the time of manuscript preparation.

## Conclusion

PE occurring approximately two hours after major gynecologic surgery can progress rapidly to obstructive shock and circulatory collapse before definitive diagnosis is established. In this patient, VA-ECMO provided a critical window for stabilization when conventional reperfusion strategies were constrained by substantial postoperative bleeding risk. The favorable outcome was achieved not only through timely extracorporeal support but also through coordinated multidisciplinary management, which enabled careful balancing of circulatory rescue, bleeding control, anticoagulation, and treatment of subsequent complications.

## Data Availability

The raw data supporting the conclusions of this article will be made available by the authors, without undue reservation.
